# CXCR4 intracellular protein promotes drug resistance and tumorigenic potential by inversely regulating the expression of Death Receptor 5

**DOI:** 10.1038/s41419-021-03730-8

**Published:** 2021-05-08

**Authors:** Mushtaq A. Nengroo, Shrankhla Maheshwari, Akhilesh Singh, Ayushi Verma, Rakesh K. Arya, Priyank Chaturvedi, Krishan K. Saini, Anup K. Singh, Abhipsa Sinha, Sanjeev Meena, Annapurna Gupta, Anjali Mishra, Jayanta Sarkar, Dipak Datta

**Affiliations:** 1 Division of Cancer Biology, CSIR-Central Drug Research Institute (CDRI), Lucknow, 226031 India; 2 Academy of Scientific and Innovative Research, New Delhi, India; 3 Sanjay Gandhi Post Graduate Institute of Medical Sciences (SGPGIMS), Lucknow, 226014 India

**Keywords:** Cancer, Cancer therapeutic resistance, Colon cancer, Cancer

## Abstract

Chemokine receptor CXCR4 overexpression in solid tumors has been strongly associated with poor prognosis and adverse clinical outcome. However, blockade of CXCL12-CXCR4 signaling axis by inhibitors like Nox-A12, FDA approved CXCR4 inhibitor drug AMD3100 have shown limited clinical success in cancer treatment. Therefore, exclusive contribution of CXCR4-CXCL12 signaling in pro-tumorigenic function is questionable. In our pursuit to understand the impact of chemokine signaling in carcinogenesis, we reveal that instead of CXCR4-CXCL12 signaling, presence of CXCR4 intracellular protein augments paclitaxel resistance and pro-tumorigenic functions. In search of pro-apoptotic mechanisms for CXCR4 mediated drug resistance; we discover that DR5 is a new selective target of CXCR4 in breast and colon cancer. Further, we detect that CXCR4 directs the differential recruitment of transcription factors p53 and YY1 to the promoter of DR5 in course of its transcriptional repression. Remarkably, inhibiting CXCR4-ligand-mediated signals completely fails to block the above phenotype. Overexpression of different mutant versions of CXCR4 lacking signal transduction capabilities also result in marked downregulation of DR5 expression in colon cancer indeed confirms the reverse relationship between DR5 and intracellular CXCR4 protein expression. Irrespective of CXCR4 surface expression, by utilizing stable gain and loss of function approaches, we observe that intracellular CXCR4 protein selectively resists and sensitizes colon cancer cells against paclitaxel therapy in vitro and in vivo. Finally, performing TCGA data mining and using human breast cancer patient samples, we demonstrate that expression of CXCR4 and DR5 are inversely regulated. Together, our data suggest that targeting CXCR4 intracellular protein may be critical to dampen the pro-tumorigenic functions of CXCR4.

## Introduction

Cancer is the second leading cause of death globally and was responsible for an estimated 9.6 million deaths in 2018^1^. Drug resistance or inadequate chemotherapy response in patients is one of the pivotal reasons for cancer-associated mortality and morbidity. Therefore, there is a dire need to understand the molecular mechanisms lying behind cancer therapy resistance. Cancer cells become therapy resistant by smartly evading the execution of apoptosis posed by chemotherapeutic drugs^2,3^. The evasion of apoptosis by cancer cells largely relies on tilting the balance towards the increased expression of anti-apoptotic genes against pro-apoptotic genes^4,5^. Death Receptors (DR) are critical pro-apoptotic factors present in healthy cells and regulate cell death. We and others have shown that paucity of death receptors in cancer cells promotes therapy resistance in diverse solid tumors^6–11^. So, the restoration of the functional death receptors is an attractive strategy to sensitize cancer cells against chemotherapy.

Over the last decade or so, chemokine receptor CXCR4 has been extensively reported to be overexpressed in most human solid tumors, and its association is strongly correlated with poor prognosis and adverse clinical outcomes^12,13^. CXCR4 ligand (CXCL12) mediated signals are well established to explain their enormous role in modulating organ-specific metastasis of solid tumors^14–16^. Still, its effect on other pro-tumorigenic functions like therapy resistance and tumor-initiating capabilities are poorly understood. Moreover, multiple inhibitors of the CXCL12-CXCR4 axis, such as AMD3100 or plerixafor, and Nox-A12 have shown limited clinical success in cancer treatment^17^. These observations pose a serious concern regarding the contribution of the CXCL12-CXCR4 signaling axis in the pro-tumorigenic role of CXCR4. Earlier, we and others have extensively reviewed the dogma and opined that CXCR7, another CXCL12 high-affinity binding receptor could be the reason for the limited success of the CXCR4 blockade in clinics^12,17–20^. Unfortunately, recent preclinical data for blocking both the chemokine receptors also failed to explain the pro-tumorigenic functions of these axes. In our relentless effort to understand the role of chemokine receptor signaling inhibition in cancer therapy^18,21–24^, here we revealed that neither CXCR7 nor CXCR4-CXCL12 signaling axis is responsible for therapy resistance and tumorigenic potential, rather CXCR4 intracellular protein in the cancer cells plays critical role in positively modulating pro-tumorigenic functions such as therapy resistance in breast and colon cancers. Our intricate in vitro and in vivo experiments in multiple colon cancer settings for CXCR4 gain and loss of function studies demonstrate that CXCR4 intracellular protein but not its CXCL12 mediated signals modulate chemotherapy resistance and tumorigenic potential via inversely regulating the expression of DR5. CXCR4-DR5 inverse regulation was also validated in diverse human cancer cell lines and human breast cancer patient samples, suggesting its possible therapeutic implications.

## Material and methods

### Reagents and antibodies

Doxorubicin, Paclitaxel, 5-fluorouracil, DAPI, anti-β-Actin (cat# A3854) antibody, Crystal violet dye, Lipophilic DiL, and DiO dyes, Bafilomycin, MG-132, Doxycycline hyclate and Polybrene were obtained from Sigma Aldrich. Cisplatin was purchased from CADILA pharmaceuticals. ImmEdge pen (hydrophobic barrier pen), Bloxall blocking solution, were purchased from Vector Laboratories, Inc. Burlingame. Fluorochrome conjugated secondary antibodies, Accutase, ProLong™ Gold Antifade Mountant, and Verso cDNA synthesis kit, as well as SYBR® Green RT-qPCR (reverse transcription-quantitative PCR) Master Mix, was purchased from Molecular Probes-Invitrogen. Taqman probes were purchased from Thermo Fischer scientific. DR5 (cat# 8074), YY1 (cat# 2185), p53 (cat# 2527), Sp-1 (cat# 9389), Cleaved Caspase-8 (cat# 9496), Cleaved-PARP (cat# 5625), p-ERK (cat# 9101), p-AKT (cat# 9271) and GAPDH (cat# 2118) antibodies were procured from Cell Signaling Technology, Inc. CXCR4 antibody for western blotting was purchased from Abcam (cat# ab124824). HRP-conjugated secondary antibodies were obtained from Santa Cruz Biotechnology. APC conjugated CXCR4, CXCR7, PE-conjugated DR5, IgG antibodies and FITC conjugated Annexin-V were purchased from BD Biosciences. Non conjugated CXCR4, CXCR7 monoclonal antibodies, chemokine CXCL12, PE-conjugated EpCAM (CD326) antibody and CXCR4 antagonist peptide AMD3100 were procured from R&D systems. All chemicals and antibodies were obtained from Sigma unless specified otherwise.

### Cell Culture

Human breast cancer cell lines MCF7, MDA-MB-468, colon cancer cell lines DLD1, HCT 116, HT 29, COLO 205, SW 620, lung cancer cell lines A549, NCI-H-358 and prostate cancer cell line PC3 were obtained from American Type Culture Collection (ATCC), USA. Mycoplasma-free early passage cells were resuscitated from liquid nitrogen vapor stocks and inspected microscopically for stable phenotype before use. MCF7, MDA-MB-468, DLD1, HT 29, COLO 2O5, A549, NCI-H-358, and PC3 cells were cultured in RPMI 1640 medium containing 10% fetal bovine serum (Gibco/Invitrogen), supplemented with anti-anti (Invitrogen). SW 620 cells were cultured in DMEM medium containing 10% fetal bovine serum (Gibco/Invitrogen), supplemented with anti-anti (Invitrogen). HCT116 cell line was cultured in McCoy’s medium containing 10% fetal bovine serum (Gibco/Invitrogen), supplemented with anti-anti (Invitrogen). Most of the cell lines widely used in the study are authenticated by STR profiling.

### Cytotoxicity assay (SRB assay)

In vitro cytotoxic activities of Doxorubicin, Paclitaxel, 5-Fluorouracil, Cisplatin, TRAIL, and AMD3100 were assessed by using standard SRB assay as described before^25,26^. The absorbance of the treated and untreated cells was measured on a multi-well scanning spectrophotometer (Epoch Microplate Reader, Biotek, USA) at a wavelength of 510 nm. Percent cell viability was calculated by taking the difference between the treated cells and the untreated cells at time 0 and multiplied by 100.

### Western blotting

Cells were lysed in RIPA buffer containing phosphatase and protease inhibitor cocktail. Protein concentration was estimated by using the BCA kit. Equal amounts of protein were resolved by SDS-PAGE and transferred to a PVDF membrane^21^. Membranes were blocked with 5% nonfat dry milk or 5% BSA followed by incubation with appropriate dilutions (1:1000) of primary antibodies overnight at 4 °C and subsequently incubated with a 1:5000 dilution of horseradish peroxidase-conjugated secondary antibodies for 1 h at room temperature. Immunoreactivity was detected by enhanced chemiluminescence solution (Immobilon^TM^ western, Millipore, USA) and scanned by the gel documentation system (Bio-Rad chemidoc XRS plus) while ImageJ software (NIH) was used for analysis and quantification. To detect CXCR4 in western blot, we followed separate sample preparation as per manufacturer’s instruction.

### Flow cytometry

Cell surface expression of CXCR4, CXCR7, and DR5 in different cell lines was analyzed by Flow cytometry. In brief, cells were allowed to grow up to 70–80% confluence and then harvested with TrypLE (Invitrogen) for single-cell suspension in FACS buffer (PBS with 0.1% BSA). Cells were stained with fluorochrome-conjugated antibodies in FACS buffer for 30–45 min at room temperature in the dark. After washing and centrifugation, cell pellets were resuspended in FACS buffer and analyzed by FACS Calibur (BD). Acquired data were analyzed using FlowJo software (Treestar). Cell sorting was carried out by FACS Aria (BD).

### DiL DiO Staining

For staining the lipophilic dye DiL (1,1’-Dioctadecyl-3,3,3’,3’ Tetramethylindocarbocyanine Perchlorate) and DiO (3,3’-Dioctadecyloxacarbocyanine Perchlorate), cells were stained with 10 μM of DiL (Red), and DiO (Green) dyes solution respectively for 45 min at 37 °C. After that, cells were spun down, rinsed, and resuspended in fresh medium and seeded in 12-well plates. The next day, cells were treated with the chemotherapeutic agents at the desired concentration and visualized under the microscope at different timings or harvested for the analysis in FACS Calibur. For FACS analysis, DiL and DiO stained cells were harvested, washed, centrifuged, and re-suspended in FACS buffer for analysis. Cells were analyzed under FL3 and FL1 channels in FACS Calibur.

### Apoptosis antibody array analysis

Apoptosis array was performed by using Proteome Profiler Human Apoptosis Array Kit (ARY009) from R&D Systems following the manufacturer’s instructions. The detailed assay procedure was followed, as described before^24^. The images were captured by the gel documentation system (Bio-Rad chemidoc XRS plus), while ImageJ software (NIH) was used for analysis and quantification. Plotly software was used for heatmap generation (Montreal, Canada).

**Site-directed mutagenesis**

Q5 site-directed mutagenesis kit (Cat# E0552S) from NEB was used to perform site-directed mutagenesis. The primers were designed from NEBase Changer online software. Primers used for L86P substitution mutation were: Forward 5ʹ GGCCGACCTCCCTTTTGTCATCACG 3ʹ; Reverse 5ʹ ACTGACAGGTGCAGCCTG 3ʹ. Primers used for deletion mutation (amino acid residues 242–248) were: Forward 5ʹ TTCGCCTGTTGGCTGCCT 3ʹ; Reverse 5ʹTGTGGTCTTGAGGGCCTTG 3ʹ. The mutation status was confirmed by sanger sequencing (Supplementary data).

### Generation of stable cell lines

CXCR4 (cat#CXCR400000), CXCR7 (cat#CXCR700000), or empty vector pcDNA 3.1 (cat# V790-20) were procured from cDNA Resource Center. For stable overexpression of chemokine receptors CXCR4 and CXCR7, MCF7 cells were plated and transfected with either overexpression plasmids or empty vector individually by using Lipofectamine LTX as transfection reagent (Invitrogen). After 48 h of transfection, cells were cultured in the presence of suboptimal dose (600 µg/ml) of Geneticin (G418) for 15 days with refreshing the medium at every third day for the selection of vector containing cells. The expression level for CXCR4 and CXCR7 was evaluated through flow cytometry and western blot.

### Generation of stable cell lines by lentiviral transduction

3rd generation lentiviral vector pUltra-Chili-Luc (addgene no. 48688) with the bi-cistronic expression of tdTomato and Luciferase was used to make HT29 cells fluorescent. Lentiviral particles were generated in HEK-293T cells. Transduction was carried out in the presence of Polybrene (8 μg/ml). A population of transduced cells (HT29-Chili-Luc) was identified by chili red expression and sorted by flow cytometry.

CXCR4 shRNA Sequence: 5ʹCCGGTCCTGTCCTGCTATTGCATTACTCGAGTAATGCAATAGCAGGACAGGATTTTTG3ʹ was cloned into the 3rd generation transfer plasmid pLKO.1 TRC cloning vector (Addgene cat no. 10878) between unique AgeI and EcoRI sites downstream of the U6 promoter. HEK-293T cell line was used for the generation of lentiviral particles using the transfection reagent Lipofectamine 2000. The media containing the viral particles was supplemented with Polybrene (8 μg/ml) for the transduction purpose. Cells were subjected to puromycin selection after 48 h of transduction, and the knockdown profile of CXCR4 was quantified after six days of selection via Flow cytometry.

### Generation of stable doxycycline-inducible CXCR4 overexpression HCT-116 cells via lentiviral transduction

CXCR4 sequence was amplified and cloned into the doxycycline-inducible pTRIPZ vector between Age-I and Mlu-I restriction sites that has a puromycin selection cassette. HEK-293T cell line was used for the generation of lentiviral particles using the transfection reagent Lipofectamine 2000. The media containing the viral particles was supplemented with Polybrene (8 μg/ml) for the transduction purpose. Cells were subjected to puromycin selection after 48 h of transduction.

### Reverse transcription-real time PCR (RT-qPCR)

Total RNA was isolated from the cultured cells and tissues using the standard procedure of the RNeasy Mini Kit (Qiagen, cat no.74104). The concentration and purity of the RNA samples were determined using nanodrop. Total RNA (5 μg) of each sample was reverse-transcribed (RT) with random hexamer according to the manufacturer’s protocol (Verso cDNA synthesis kit). The final cDNA was diluted with nuclease-free water (1:3), 1 μl of this having a concentration of 25 ng/μl was used for each reaction in real-time PCR. Real-time PCR was carried out using an ABI StepOnePlus Real-Time PCR System (Applied Biosystems). TaqMan gene expression assay from Thermo Fisher Scientific was used for CXCR4 (Assay ID: Hs00607978_s1), and DR5 (Assay ID: Hs00366278_m1) gene amplification. Reactions for each sample were performed in triplicate. GAPDH or 18 s amplification was used as the housekeeping gene. Standard delta-delta Ct method is used to calculate the relative fold change in gene expression. For amplifying YY1, p53, Sp1, we performed SYBR Green-based RT-PCR following manufacturer’s instructions.

### Chromatin immunoprecipitation (ChIP)

ChIP assay was conducted by using the ChIP assay kit (Cell Signaling Technology) following the manufacturer’s protocol. In brief, cells at 80% confluency were fixed with formaldehyde (1% final concentration directly to the culture media) for 10 min. Cells were then centrifuged, followed by lysis in 200 μl of membrane extraction buffer containing protease inhibitor cocktail. The cell lysates were digested with MNase for 30 min at 37 ˚C to get chromatin fragments followed by sonication (with 20 s on/20 s off 3 Sonication cycles at 50% amplitude) to generate 100–500 bp long DNA fragments. After centrifugation, clear supernatant was diluted (100:400) in 1X ChIP buffer with protease inhibitor cocktail followed by keeping 10% of input control apart and incubated with primary antibody or respective normal IgG antibody overnight at 4 ˚C on a rotor. The next day, IP reactions were incubated for 2 h in ChIP-Grade Protein G Magnetic Beads, followed by precipitation of beads and sequential washing with a low and high salt solution. Then elution of chromatin from Antibody/Protein G Magnetic beads and reversal of crosslinks by using heat was carried out. DNA was purified by using spin columns, and SYBR Green real-time PCR was conducted. Primer sequences used for the ChIP experiment for different genes are as follows:

Human YY1 on DR5 repressor site, Forward: 5ʹ-TGGTTCCACACATCCCTGAA-3ʹ, Reverse: 5ʹ-CGCAAGCAGAAAAGGAGGTC-3ʹ, Human p53 on DR5, Forward: 5ʹ-CTCGAGGTCCTGCTGTTGGTGAGT-3ʹ, Reverse: 5ʹ-GAGCTCGGGAATTTACACCAAGTGGAG-3ʹ.

### Immunofluorescent staining and confocal microscopy

Tumor samples resected from breast cancer patients obtained from the Department of Endocrine Surgery at the SGPGI, Lucknow, after having due Institutional ethical clearance. Part of it was fixed in 4% paraformaldehyde for 48 h and embedded in paraffin wax. The staining of sections was performed as per the manufacturer’s recommendations (Vector Laboratories). Tissue sections were de-paraffinized and rehydrated in graded alcohol. Antigen retrieval was performed by immersing slides in 10 mM sodium citrate buffer (pH 6) and boiled in a high-power microwave oven for 25 min. Tissue sections were then washed with PBS followed by 25-min incubation in bloxall to neutralize endogenous peroxide activity and subsequently incubated with primary monoclonal antibodies against CXCR4 (1:800) and DR5 (1:400) in 2% BSA overnight at 4 °C. The next day, the appropriate fluorochrome-conjugated secondary antibody was added and incubated for one hour. The tissue sections were then mounted by using antifade (GIBCO). Stained sections were observed under a confocal microscope (Zeiss Meta 510 LSM; Carl Zeiss). It is necessary to mention that detecting DR5 in tissue sections is challenging due to the poor availability of compatible DR5 antibodies. We tried multiple antibodies for DR5 staining in tumor tissue sections, and the best one is represented in the figure.

For confocal microscopy, cells were seeded and grown on coverslips and treated with or without doxycycline for 48 h. After intermediate washes with cold PBS, cells were fixed with 4% paraformaldehyde, permeabilized by 0.1% NP40, followed by blocking with 2% BSA. After overnight incubation with primary antibodies at 4 ˚C, cells were washed and incubated with fluorescent-conjugated appropriate secondary antibodies followed by DAPI staining. After washing, cells were mounted on glass slides and analyzed under an inverted Zeiss confocal laser scanning microscope (Zeiss meta 510 LSM; Carl Zeiss).

### In vivo studies in xenograft tumor models

All animal studies were conducted by following standard principles and procedures approved by the Institutional Animal Ethics Committee (IAEC) of CSIR-Central Drug Research Institute. All the animals were maintained in a pathogen-free facility under a day–night cycle. Following our well-established colon cancer xenograft models as described earlier^27^, 2 × 10^6^ or 5 × 10^6^ cells (HT-29, DLD1, and HCT-116 control, CXCR4 KD, or Doxycycline-inducible CXCR4 Overexpression) in 100 μl PBS were subcutaneously inoculated into the flanks of the left/and or right hind leg of each 4–6 weeks old nude Crl: CD1-Foxn1^nu^ mice or NOD/SCID mice. Mice were randomly assigned to groups by a blinded independent investigator. Throughout the study, the tumor was measured with an electronic digital caliper at a regular interval, and the tumor volume was calculated using standard formula V = Π/6 × a^2^ × b, where ‘a’ is the short and ‘b’ is the long tumor axis. At the end of the experiment, mice were sacrificed, and subcutaneous tumors were dissected for further studies.

Parts of harvested tumors were minced into small pieces with sterile forceps and scissors in the laminar hood. These pieces of tumors are digested with collagenase, and the cells were passed through a cell strainer of pore size 70 micron to get single cells. Isolated single cells from tumors were either used for FACS staining or cultured under puromycin selection for further experiments.

### Analysis of GDC TCGA Breast Cancer (BRCA) dataset

Illumina HiSeq mRNA data of Breast Cancer Cell Lines (Heiser 2012) and patients with breast cancer, GDC TCGA Breast Cancer (BRCA), was downloaded from the TCGA portal for CXCR4 and *DR5* genes using https://xena.ucsc.edu/ browser^28^. Log2 (fpkm-uq + 1) values for CXCR4 and DR5 were converted into fold changes and compared to identify the association between CXCR4 and *DR5* genes. Online software Heatmapper was used for the heatmap generation, and the clustering method used was Average Linkage, whereas the Euclidean distance measurement method was considered.

### Statistics

Most of the in vitro experiments are representative of at least three independent experiments or specified otherwise in the figure legends. Two-tailed Student’s *t* test was used to examine statistically significant difference for two-group analysis. All data are presented as means ± SEM. These analyses were done with Graph-Pad Prism software. Results were considered statistically significant when *p* values ≤ 0.05 between groups.

## Results

### CXCR4 but not CXCR7 regulates paclitaxel resistance in cancer

**Fig. 1:**
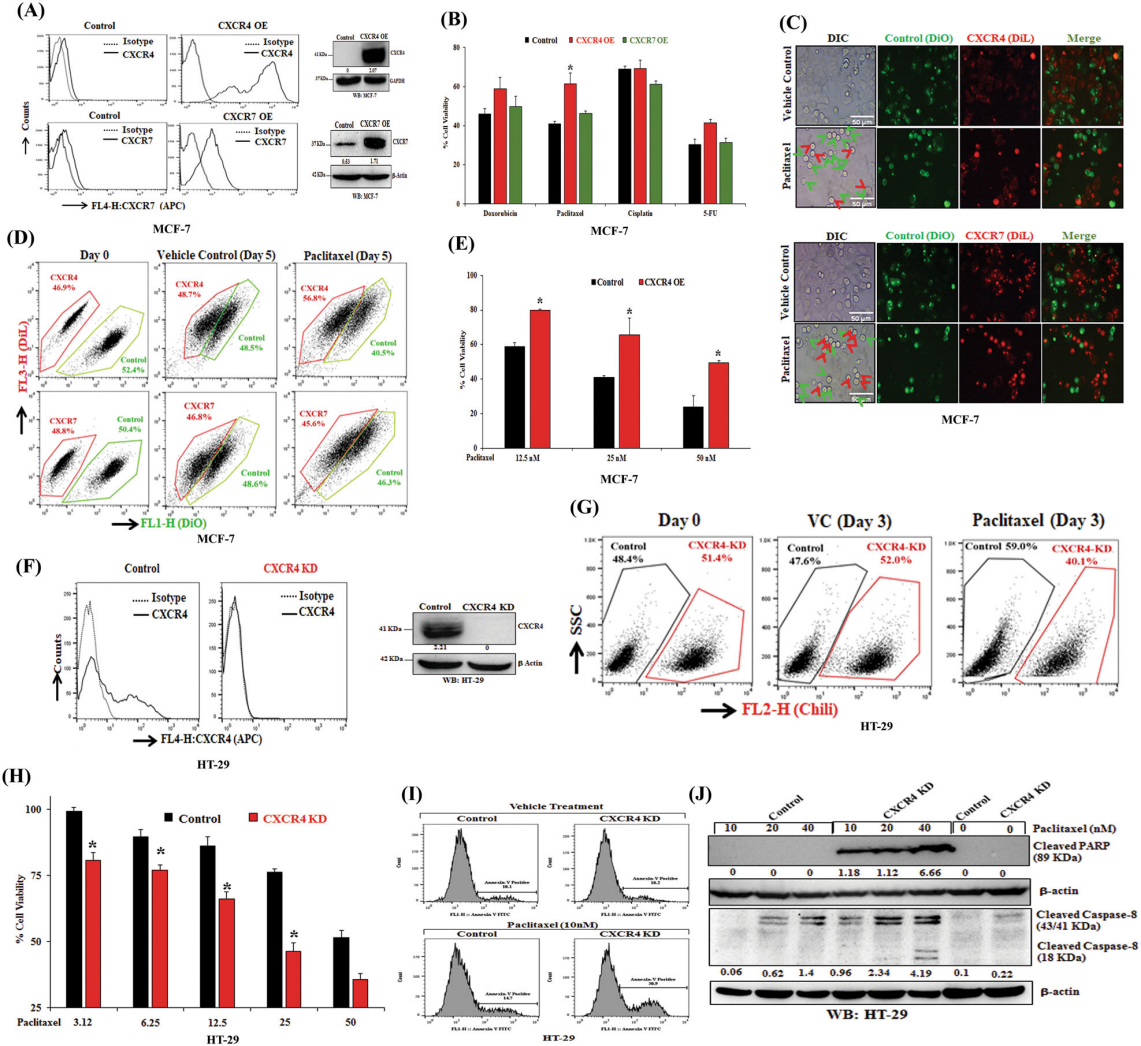
CXCR4 regulates paclitaxel resistance in cancer. **A** MCF7 cells were transfected with either empty vector pcDNA3.1 or vector containing the gene for the overexpression of either chemokine receptor CXCR4 or CXCR7 and made stable. These stable cell lines were stained with either APC-conjugated anti-human CXCR4 (CD184) or anti-human CXCR7 antibodies along with their respective isotype control antibodies and analyzed by flow cytometry. The surface expression levels of CXCR4 and CXCR7 are represented in histogram overlays (left panel). Western blot analysis of CXCR4 and CXCR7 in the lysate of control and CXCR4/CXCR7 overexpression MCF-7 cells; GAPDH and β-Actin were used as the protein loading control (right panel). **B** CXCR4 or CXCR7 overexpressing and control MCF7 cells were treated with Doxorubicin (250 nmol/L), Paclitaxel (25 nmol/L), Cisplatin (2.5 μmol/L), or 5-Fluorouracil (25 μmol/L) for 72 h and cytotoxicity was measured by SRB assay as described in materials and methods. Percent cell viability was tabulated. Columns, an average of triplicate readings of samples; error bars ± SEM. *p < 0.05, compared with control cells. **C** DiL (red) stained CXCR4 or CXCR7 overexpressing MCF7 cells and DiO (green) stained control MCF7 cells were mixed in equal numbers, seeded in 6-well plate and treated with vehicle or Paclitaxel (10 nmol/L) for 3 days and then analyzed via fluorescence microscopy; red arrows indicate DiL stained dead CXCR4 or CXCR7 overexpressing cells, while green arrows indicate DiO stained dead control cells. Photomicrographs are representative of three independent experiments. **D** DiL stained CXCR4 overexpressing or CXCR7 overexpressing MCF7 cells were equally mixed with DiO stained control MCF7 cells, and a small aliquot of mixture was acquired as day 0 reading by FACS. The rest of the cells were treated either with vehicle or Paclitaxel (10 nmol/L) for 5 days and subsequently analyzed by FACS. Data are representative of at least three independent experiments. **E** CXCR4 overexpressing and control MCF7 cells were treated with different concentrations of paclitaxel (12.5, 25, 50 nM) for 48 h, and cytotoxicity was evaluated by SRB assay. Percent cell viability was tabulated. Columns, an average of triplicate readings of samples; error bars ± SEM.*p < 0.05, compared to control cells treated with respective doses of paclitaxel. **F** Chili tagged HT-29 cells were made stable for the knockdown of CXCR4 via shRNA mediated lentiviral transduction; scramble shRNA transduced stable HT-29 cells were used as control. CXCR4 knockdown and control HT-29 cells were stained either with APC-conjugated anti-human CXCR4 (CD184) antibody or with appropriate isotype control antibody and analyzed by FACS. Histogram overlays represent the cell surface expression of CXCR4 (left panel). Western blot analysis of CXCR4 in the lysate of control and CXCR4 knockdown HT29 cells; β-Actin was used as the protein loading control (right panel). **G** Control and CXCR4 knockdown Chili tagged HT29 cells were mixed equally and analyzed by FACS either at day 0 or after three days of vehicle/paclitaxel (20 nmol/L) treatments. **H** CXCR4 knockdown and control HT-29 cells were treated with different concentrations of Paclitaxel (3.12, 6.25, 12.5, 25, 50 nM) for 48 h and cytotoxicity was measured by SRB assay. Percent cell viability was tabulated. Columns, an average of triplicate readings of samples; error bars ± SEM.*p < 0.05 compared to control cells. **I** Control and CXCR4 knockdown HT29 cells were treated with paclitaxel for 24 h and stained with FITC conjugated Annexin-V. Histogram overlays show the Annexin-V positive cells. **J** Western blot analysis of cleaved PARP and Caspase-8 in the lysate of 24 h post vehicle or Paclitaxel treated (10, 20, and 40 nM) control and CXCR4 knockdown HT-29 cells; β-Actin was used as the protein loading control. Western Blot densitometric quantification numbers are shown above the loading control blot of all immunoblot studies.

CXCL12 acts as a ligand for chemokine receptor CXCR4 and CXCR7^12,14,29^, and these signaling axes have shown to be hyper activated in cancer with poor clinical outcome^30,31^. Here, we sought to determine the impact of CXCR4 and CXCR7 on chemotherapy resistance. We selected the MCF-7 cell for overexpression studies as it showed very negligible surface expression of both CXCR4 and CXCR7. After confirming stable CXCR4 and CXCR7 overexpression in MCF-7 cells compared to vector control as shown by FACS analysis and Western blot (Fig. 1A), the cells were treated with FDA approved anticancer drugs like Doxorubicin (250 nM), Paclitaxel (25 nM), Cisplatin (2.5 μM), and 5-Fluorouracil (25 μM) and cytotoxic effects of these drugs were assessed. As observed in Fig. 1B, CXCR4 overexpressed MCF-7 cells significantly (*p* < 0.05), resists paclitaxel-induced cell death compared to control cells. Further, CXCR4 overexpressed cells show some degree of resistance against Doxorubicin and 5-FU, whereas similar kind of response was missing in the case of CXCR7 overexpressing cells. Next, to validate the impact of CXCR4 or CXCR7 high expression on the drug-resistant property of the cancer cells, we stained control MCF7 cells with DiO (green dye) and CXCR4 or CXCR7 overexpressing cells with DiL (red dye) respectively^32^. We mixed and seeded an equal number of DiO stained control cells with DiL stained CXCR4 or CXCR7 overexpressing cells and treated with vehicle control (DMSO) or paclitaxel (10 nmol) for 3 or 5 days and then monitored under either fluorescence microscopy (Fig. 1C) or flow cytometry (Fig. 1D) respectively. As shown in Fig. 1C. The fewer number of dead CXCR4 overexpressing cells as compared to others indicate that CXCR4 contributes markedly to paclitaxel resistance. In our quantitative flow cytometry analysis, we observed that CXCR4 overexpressing MCF7 cells were higher in number than control cells, whereas there was no substantial change in the number of CXCR7 overexpressing cells as compared to control cells (Fig. 1D). Next, we observed gain of CXCR4 function pose significant resistance to cell death for all three doses (12.5, 25, 50 nM) of paclitaxel tested (Fig. 1E). Therefore, these extensive experimental validations demonstrate that selective CXCR4 gain of function but not CXCR7 overexpression result in chemotherapy, especially paclitaxel resistance in breast cancer.

Now, we wanted to test the impact of the loss of function of CXCR4 on paclitaxel-induced cytotoxicity. HT-29 cells were selected for knockdown studies as it has been shown to express CXCR4 robustly at the basal level. HT-29 cells were made stable for CXCR4 knockdown via shRNA-mediated lentiviral transduction (Fig. 1F). After paclitaxel treatment for three days, Chili-Luc HT-29 (CXCR4 knockdown) cells were substantially low in number (40.1%) as compared to control cells (59.0%) (Fig. 1G). Next, to test the cell growth inhibitory effect of paclitaxel against CXCR4 knockdown HT-29 cells, we performed standard SRB assay and observed that paclitaxel treatment significantly (*p* < 0.05) induces cytotoxicity in CXCR4 knockdown HT-29 cells as compared to control cells (Fig. 1H). These data together suggest that the loss of CXCR4 sensitized the cells to paclitaxel treatment. To understand the cell death mechanism involved, Annexin-V staining or immunoblotting for PARP- and Caspase-8 cleavage, after paclitaxel treatment, was performed. It was observed that there is an increase of Annexin-V staining as well as cleaved-PARP and cleaved-Caspase-8 level in CXCR4 knockdown HT-29 cells as compared to control cells under different concentrations of paclitaxel treatment, indicating CXCR4 loss of function is priming cells for paclitaxel-induced apoptosis (Fig. 1I, J).

### CXCR4 inversely regulates expression and function of DR5

**Fig. 2:**
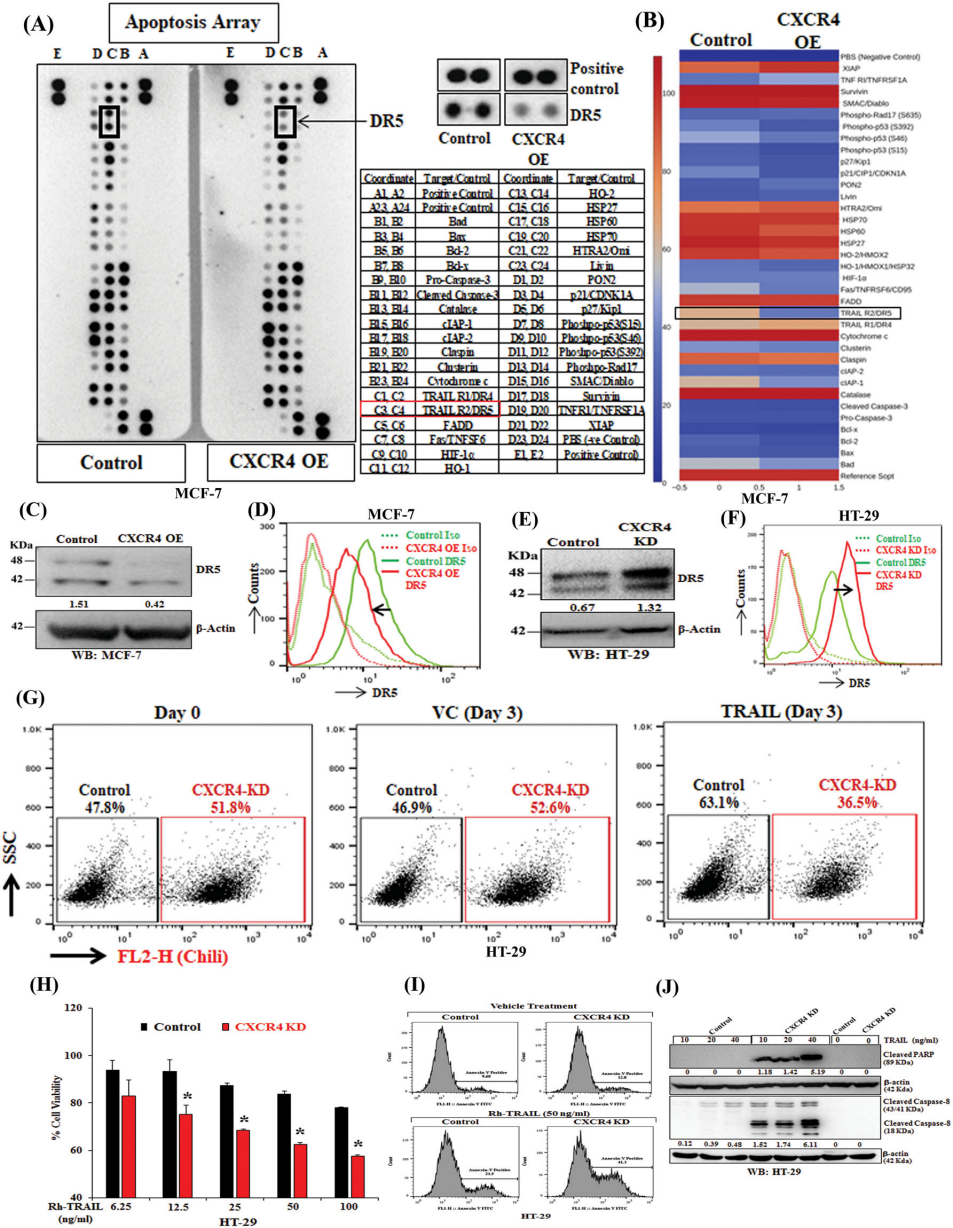
CXCR4 inversely regulates the expression and function of DR5. CXCR4 overexpressing or control stable MCF7 cells were harvested for protein extraction and analyzed for the expression of apoptotic genes by utilizing proteome profiler apoptosis array of individual western blot analysis. **A** Chemiluminescent image of the expression of 35 apoptosis-related genes with positive and negative controls in duplicates for control and CXCR4 overexpressed cells was shown (left panel). The enlarged images of selected apoptotic protein (DR5) found to be markedly altered in the proteome profiler array (middle-upper) and spot coordinates (middle-lower) were shown. **B** Heatmaps depicting differentially regulated proteins in CXCR4 overexpressing and control stable MCF7 cells. **C** Immunoblot analysis of DR5 protein in CXCR4 overexpressing or control MCF7 cells; β-Actin was used as an internal protein loading control. **D** CXCR4 overexpression and control MCF7 cells were either stained with PE-conjugated anti-human DR5 or PE tagged isotype (IgG) control antibodies, and cell surface expression of DR5 was analyzed by histogram overlays using FACS. **E** Immunoblot analysis of DR5 protein in CXCR4 knockdown or control HT-29 cells; β-Actin was used as an internal protein loading control. **F** CXCR4 knockdown and control HT-29 stable cells were stained either with PE-conjugated anti-human DR5 or PE tagged isotype control antibodies. Cell surface expression of CXCR4 was analyzed by histogram overlays using FACS. **G** Chili tagged CXCR4 knockdown and control HT-29 stable cells were mixed equally and subjected to FACS analysis at Day 0 and 3 days after recombinant human TRAIL (50 ng/mL) treatment. **H** CXCR4 knockdown and control stable cells were treated with different concentrations of TRAIL (6.25, 12.5, 25, 50, 100 ng/mL) for 48 h, and cytotoxicity was measured by SRB assay. Percent cell viability was tabulated. Columns, an average of triplicate readings of samples; error bars ± S.D. **p < 0.01; #, *p* < 0.05, compared to TRAIL-treated control cells. **I** Control and CXCR4 knockdown HT29 cells were treated with Rh-TRAIL for 24 h and stained with FITC conjugated Annexin-V. Histogram overlays show the Annexin-V positive cells. **J** Immunoblot analysis of cleaved PARP and Caspase-8 in 24 h post vehicle or TRAIL-treated (10, 20, and 40 ng/mL) control and CXCR4 knockdown HT-29 cell lysates; β-Actin was used as the protein loading control. Western Blot densitometric quantification numbers are shown above the loading control blot of all immunoblot studies.

To explore the molecular mechanism of CXCR4 mediated therapeutic resistance, apoptosis antibody array was performed where we found a very selective and marked reduction of expression of pro-apoptotic protein DR5 in CXCR4 overexpressed cells as compared to control cells, whereas, changes in other apoptotic proteins were found to be minimal (Fig. 2A, B). To validate apoptosis array results, we performed individual western blot and flow cytometry for CXCR4 overexpressed (Fig. 2C, D) and knockdown cells (Fig. 2E, F) along with their respective controls. As shown in Fig. 2C–F, CXCR4 inversely regulates total and surface protein levels of DR5 compared with their respective control cells. To examine the functional upregulation of DR5 under the CXCR4 knockdown condition, we mixed an almost equal quantity of HT-29 CXCR4 knockdown Chili-Luc cells (51.8%) and control healthy cells (47.8%), and treated with vehicle control or recombinant human TRAIL (50 ng/mL) for three days and were quantitatively analyzed through flow cytometry. As shown in Fig. 2G, CXCR4 knockdown Chili-Luc cells were comparatively less in number (36.5%) as compared to control cells (63.1%), suggesting that CXCR4 knockdown resulted in induction of functional DR5 on the cancer cell surface. Also, we observed dose-dependent (rhTRAIL) increase in cell death in CXCR4 KD cells compared to control (Fig. 2H). Further, it was observed that rhTRAIL markedly induces apoptosis by increasing Annexin-V^+^ cells and the level of cleavage of PARP and Caspase-8 in CXCR4 KD cells compared to their respective controls (Fig. 2I, J). Altogether, our data establish that CXCR4 inversely alters the expression of DR5, and loss of its function results in TRAIL-mediated sensitization of cancer cells.

### CXCR4 mediated DR5 downregulation is dependent on the recruitment of transcription factors p53 and YY1 to its promoter

**Fig. 3:**
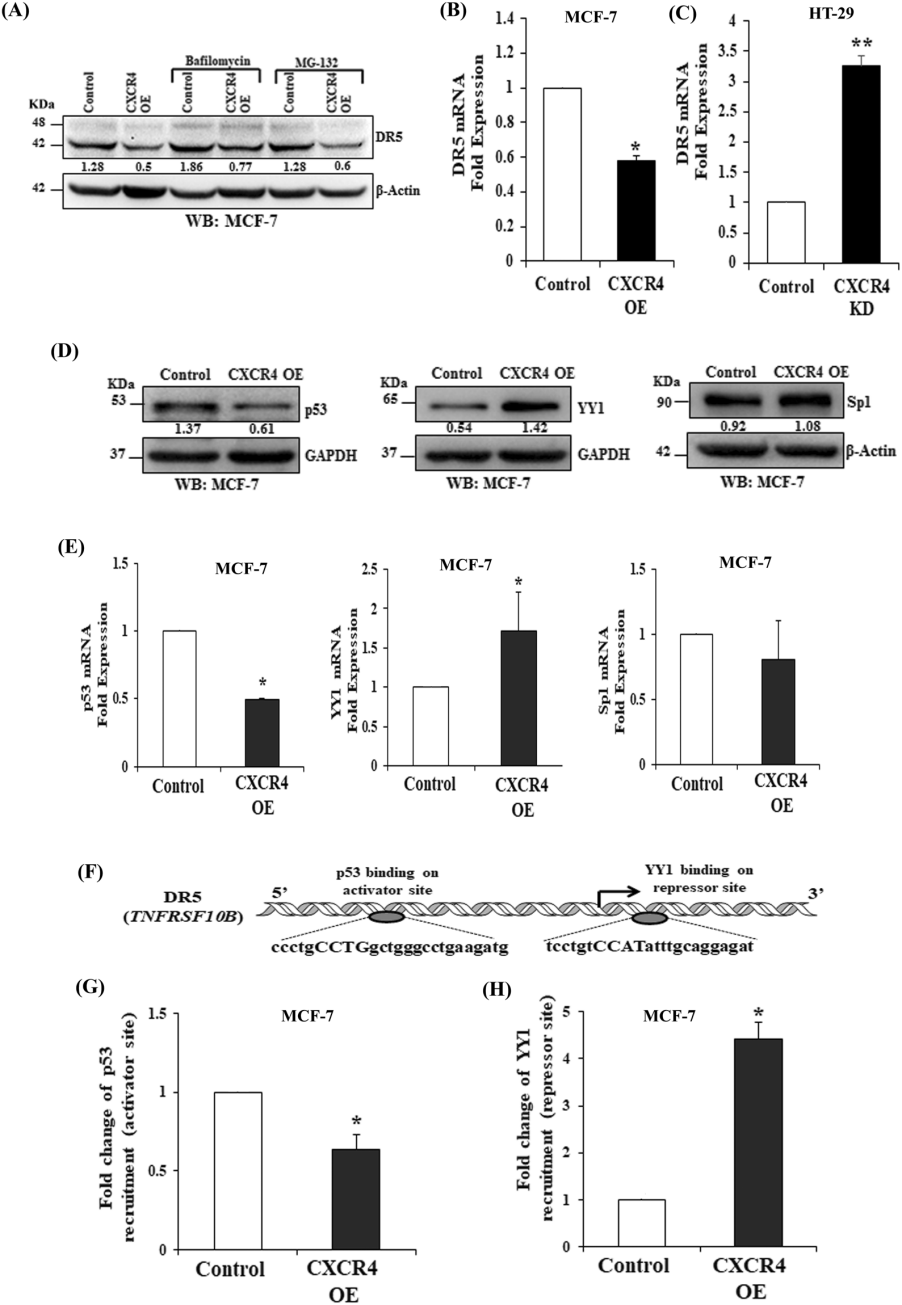
CXCR4 regulates DR5 transcription by differentially modulating the recruitment of transcription factors p53 and YY1 at the promoter site of DR5. **A** Western blot analysis of DR5 in control and CXCR4 overexpressing MCF-7 cells after bafilomycin or MG132 treatment for 5 h; β-actin was used as the protein loading control. **B**, **C** Total RNA was isolated from CXCR4 overexpressing (MCF-7) and knockdown (HT-29) stable cells along with their respective controls and reverse transcribed. Fold change in DR5 mRNA expression was measured by RT-qPCR as described in Materials and Methods. Data are representative of three independent experiments, resulting from duplicate readings of two different samples; Columns, average value of DR5 mRNA expression; bars ± SEM. *, *p* < 0.05, compared with respective controls. **D** Western blot analysis of p53, YY1, and Sp1 in control and CXCR4 overexpressing MCF-7 cells; GAPDH or β-actin was used as the protein loading control. Western Blot densitometric quantification numbers are shown above the loading control blot of all immunoblot studies. **E** Fold change in mRNA expression of p53, YY1, and Sp1 in control and CXCR4 overexpressing stable MCF-7 cells was assessed by RT-qPCR; Columns, average value of p53/yy1/sp1 mRNA expression; bars ± SEM. *, *p* < 0.05, compared with respective control. **F** Diagrammatic representation for the p53 binding on activator site as well as YY1 binding on repressor site of the DR5 gene promoter region. **G**, **H** ChIP assay for the analysis of YY1 and p53 recruitment on the DR5 gene promoter in CXCR4 overexpressing and control stable MCF7 cells followed by RT-qPCR. Fold change in p53 and YY1 recruitment on the respective activator and repressor sites of the DR5 gene promoter were assessed in control and CXCR4 overexpressing MCF-7 cells. Results are representative of at least two independent experiments; Columns, an average of duplicate readings of samples; error bars ± S.D. *p < 0.05 versus control MCF7 cells.

Next, we sought to determine the molecular mechanisms involved in CXCR4 mediated DR5 regulation. Although lysosomal and proteasomal degradation of DR5 is common in cancer cells, Bafilomycin and MG-132 inhibitors failed to markedly rescue CXCR4 mediated DR5 downregulation (Fig. 3A). Next, mRNA expression was assessed by RT-qPCR. As shown in Fig. 3B, C, *DR5* mRNA expression (TNFRSF10B) was found to be significantly (*p* < 0.05) lower in CXCR4 overexpressing cells as compared to control cells and vice-versa in knockdown cells as compared to their respective control. As p53 and Sp1 transcription factors are the positive regulators, while YY1 is a negative regulator of *DR5* gene transcription^33–35^. Further, we observed that p53 was found to be downregulated whereas; the expression of YY1 goes up under the influence of CXCR4 overexpression as compared to the controls in both MCF-7 and HCT-116 cells (Fig. 3D and Supplementary Fig. S1). Compared to control, significant (*p* < 0.05) downregulation of p53 mRNA and upregulation of YY1 mRNA was observed after CXCR4 overexpression as determined by RT-qPCR analysis (Fig. 3E). These data indicate that the DR5 regulating transcription factors, p53 and YY1, are themselves regulated at the transcriptional level in response to the overexpression of CXCR4 in cancer cells. Recently, we demonstrated H3K27me3-mediated transcriptional regulation of DR5 in colon cancer by extensive ChIP analysis^9^. Next, we performed promoter analysis for putative binding sites of YY1 and p53 on DR5 promoter by using the MatInspector tool from Genomatix software. By using the software, we found one activator binding site for p53, and one repressor binding site of YY1 on the DR5 promoter region (Fig. 3F). We designed the primers for these protein binding sites on the DR5 promoter. In our Chromatin Immunoprecipitation (ChIP) assay, we found significantly decreased binding of p53 (Fig. 3G) on the DR5 promoter and increased recruitment of YY1 (Fig. 3H) on the repressor site; in CXCR4 overexpressing cells as compared to control cells. Thus, from the overall results of immunoblot, RT-qPCR, and ChIP assays, it is clear that the downregulation of DR5 in CXCR4 overexpressing cells is mediated by the decreased recruitment of p53 on its activator site and increased recruitment of YY1 on its repressor site of the DR5 promoter region.

### CXCR4 mediated DR5 regulation is independent of CXCL12-CXCR4 signaling

**Fig. 4:**
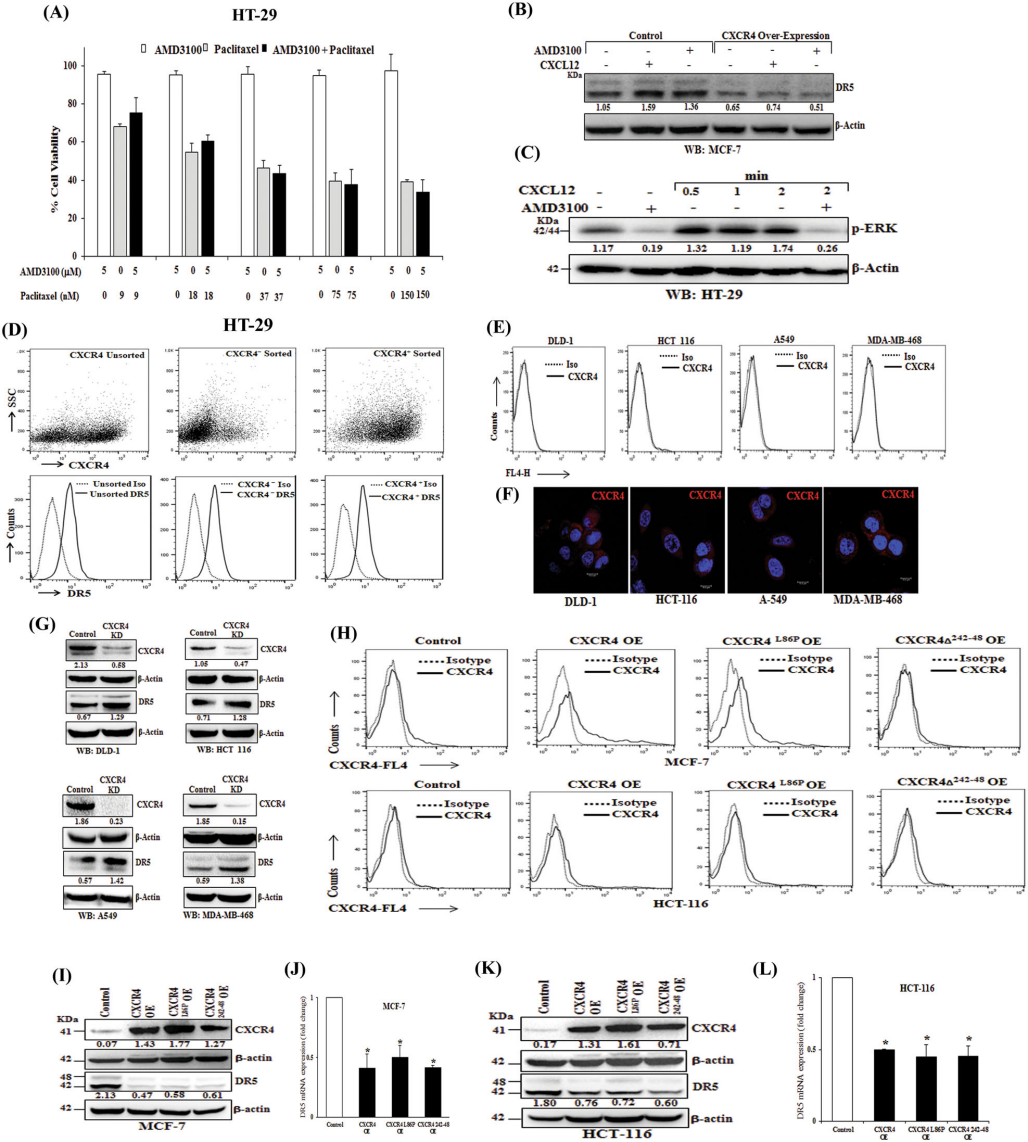
CXCR4 mediated DR5 regulation is independent of CXCR4-CXCL12 signaling. **A** HT-29 cells were treated with either different concentrations of paclitaxel (9, 18, 37, 75, 150 nM) or AMD3100 (5 μM) alone or in combinations for 48 h and cytotoxicity was measured by SRB assay. Percent cell viability was tabulated. Columns, an average of triplicate readings of samples; error bars ± SEM. **B** Control and CXCR4 overexpressed MCF-7 cells were treated with CXCR4 ligand CXCL12 (100 ng/ml) or CXCR4 antagonist AMD3100 (5 μmol/L) for 12 h, and subjected to Western blot analysis for DR5 and β-actin. **C** HT-29 cells were either pre-treated with vehicle or AMD3100 (5 μM) for 12 h, followed by treatment with CXCR4 ligand CXCL12 (100 ng/ml) for different time points (0.5, 1, and 2 mins) and subjected to Western blot analysis for p-ERK and β-actin. **D** CXCR4^+^ and CXCR4^−^ HT-29 cells were flow-sorted and plated. After 5 days of culture, cells were stained with either APC-conjugated CXCR4 (CD184) and PE-conjugated DR5 or their respective matched isotype control antibodies and analyzed by FACS. In the upper panel, dot plots represent CXCR4 staining in unsorted, CXCR4^+^sorted and, CXCR4^−^ sorted cells. In the lower panel histograms represent DR5 staining in the above-mentioned respective cells. **E** DLD-1, HCT-116, A-549, and MDA-MB-468 cells were stained with either APC-conjugated anti-human CXCR4 (CD184) or isotype control antibodies and analyzed by FACS. The cell surface expression of CXCR4 is represented in histogram overlays. **F** DLD-1, HCT-116, A-549, and MDA-MB-468 cells were seeded on coverslips for 24 h and subjected to immunofluorescence staining for CXCR4 and analyzed by confocal microscopy; Scale bar 10 μm. **G** DLD-1, HCT-116, A-549, and MDA-MB-468 cells were made stable for CXCR4 knockdown via shRNA mediated lentiviral transduction and scramble shRNA transduced cells were used as control. Immunoblot analysis of CXCR4 and DR5 protein in control or CXCR4 knockdown cells are shown; β-Actin was used as an internal protein loading control. **H**–**L** MCF-7 and HCT-116 cells were transfected with scrambled, wild type CXCR4, CXCR4^L86P^, or CXCR4^δ242-248^ containing vectors and cultured. After 48 h, cells were either stained with APC-conjugated anti-human CXCR4 (CD184)/isotype control antibodies and analyzed by FACS, or subjected to western blot or total RNA isolation. **H** The cell surface expression of CXCR4 is represented in histogram overlays. **I**, **K** Immunoblot analysis of CXCR4 and DR5 protein in control, wild type CXCR4, CXCR4^L86P^, or CXCR4^δ242-248^ transfected MCF-7 and HCT-116 cells; β-Actin was used as an internal protein loading control. Western Blot densitometric quantification numbers are shown above the loading control blot of all immunoblot studies. **J**, **L** Fold change in DR5 mRNA expression was measured by RT-qPCR as described in Materials and Methods. Data are representative of three independent experiments, resulting from duplicate readings of two different samples; Columns, average value of DR5 mRNA expression; bars ± SEM. *, *p* < 0.05, compared with respective controls.

Reverse regulation of cancer cell apoptosis and functional expression of pro-apoptotic protein DR5 under CXCR4 gain and loss of function encouraged us to test the contribution of CXCR4 ligand CXCL12 mediated signaling axis for delivering the above phenotype. We used FDA-approved CXCR4 antagonist drug AMD3100^36,37^ or paclitaxel alone or in combination in HT-29 cells to mimic the phenotype of our CXCR4 knockdown experiments. Surprisingly, we did not find any significant synergistic cytotoxic effect in combination treatment compared to individual treatments (Fig. 4A). Further, we treated our control and CXCR4 overexpressed MCF-7 cells either with AMD3100 or CXCL12 and sought to observe the change in suppressed DR5 expression. Compared to control, here we also did not observe any rescue (in case of AMD3100 treatment) or further suppression (CXCL12 treatment) of DR5 expression following respective treatments (Fig. 4B). Next, we observed CXCL12 mediated ERK and AKT phosphorylation was found to be markedly inhibited by AMD3100 treatment (Fig. 4C and Supplementary Fig. S2) strongly advocating the fact that both CXCL12 and AMD3100 are functional in our system and confirmed the accuracy of our previous unanticipated observations. Further, we sorted CXCR4^+^ and CXCR4^−^ cells by FACS from HT29 cells, allowed them to grow for 5 days, and analyzed the expression of DR5 on day 0 and day 5. As shown in Fig. 4D, upper and lower panels, sorted cells maintained their CXCR4^+^ and CXCR4^−^ status; however, there was no substantial difference in the expression of DR5 in the beginning or in the fifth day of culture. To further validate the above observations, we examined the surface expression of CXCR4 in several different cancer cell lines like DLD-1, HCT- 116, A-549, and MDA-MB-468, where there is negligible or no surface expression of CXCR4 (Fig. 4E), however, CXCR4 protein is present in all the cases in the cytoplasm as observed by confocal microscopy (Fig. 4F). Interestingly, CXCR4 knockdown in all the cases markedly induces the expression of DR5 across various cancer cell lines of human origin (Fig. 4G). Next, different CXCR4 overexpression constructs were created where; we either inserted single mutation or deleted critical amino acids to abolish CXCR4-ligand-mediated signals^38^. Here, we found that the expression of CXCR4 on the cell surface is differentially controlled in different cell types as MCF-7 cells are more efficient than HCT-116 in terms of transferring CXCR4 overexpressed proteins into the cell surface (Fig. 4H). Also, we observed that irrespective of fully functional (signaling competent) or signaling impaired versions of CXCR4 overexpression resulted in robust downregulation of DR5 expression at protein and mRNA level in MCF-7 and HCT-116 cells (Fig. 4I–L). Altogether, our data suggest that intracellular CXCR4 protein, but not CXCR4-CXCL12 mediated signals, regulates the expression of DR5 in cancer cells.

### Loss of CXCR4 protein results in compromised colon tumor growth in vivo and sensitized tumors against paclitaxel therapy

**Fig. 5:**
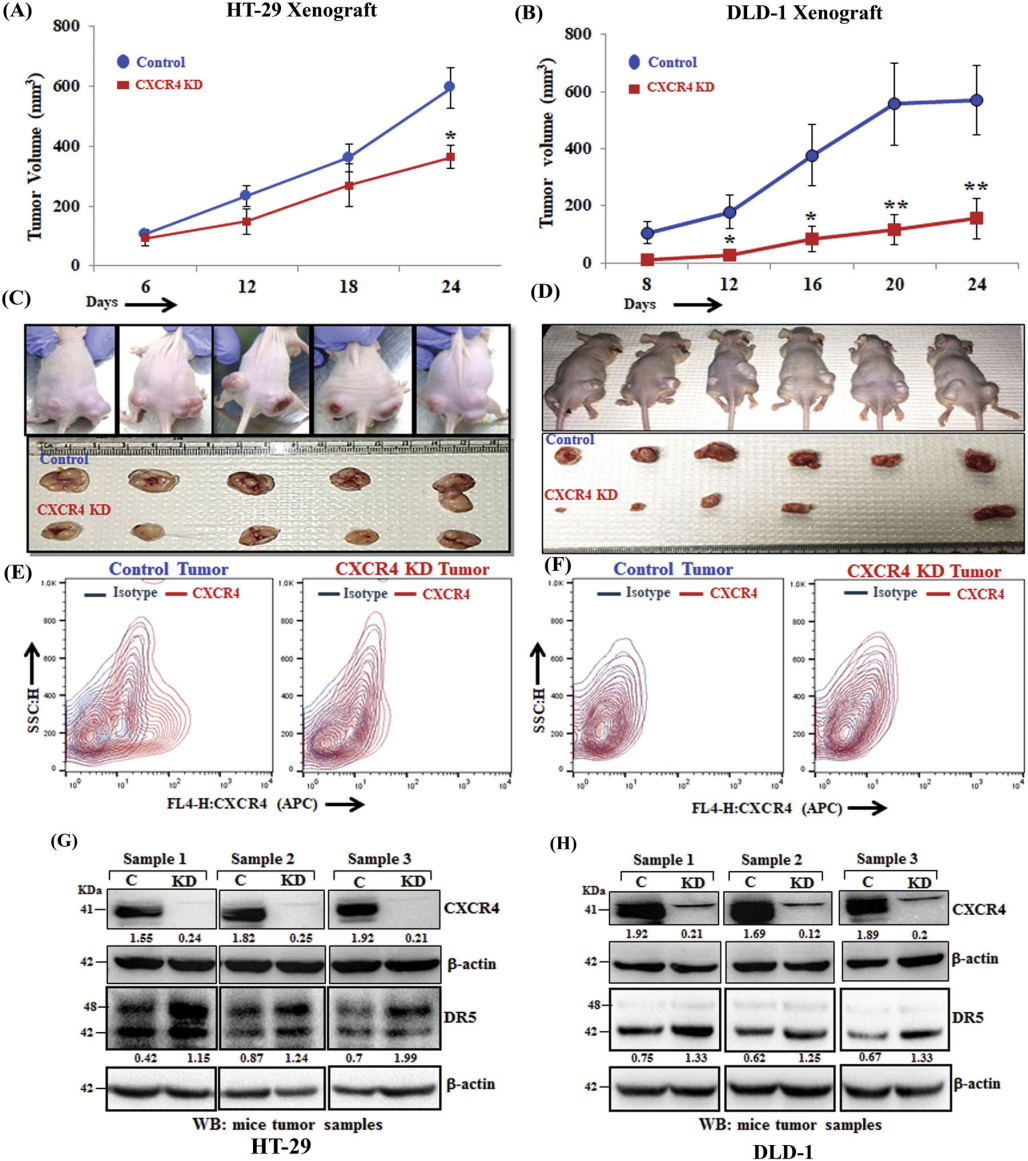
CXCR4 protein knockdown results in compromised tumor growth and DR5 overexpression in vivo. In total, 2 × 10^6^ stable control (HT-29 and DLD-1) or CXCR4 knockdown (HT-29 and DLD-1) cells in 100μl PBS were injected subcutaneously in the flanks of the right or left hind leg of 4–6 weeks old Crl: CD1-Foxn1^nu^ mice respectively. Tumor volumes were measured after regular intervals by using a caliper. Growth curves for HT-29 (**A**) and DLD-1 (**B**) are shown for tumors generated from control and CXCR4 knockdown cells; points are indicative of the average value of tumor volume ± SE (*n* = 5 for HT-29, *n* = 6 for DLD-1); *p < 0.05 compared to control tumors. **C**, **D** Upper panels represent images of tumor-bearing mice, control (right flank), and CXCR4 knockdown (left flank). Mice were sacrificed, and the respective tumors from HT29 (**C**) and DLD-1 (**D**) were harvested and shown in photographs in lower panels. Single cells from respective control and CXCR4 knockdown HT-29 (**E**) and DLD-1 (**F**) were harvested and stained with either APC-conjugated anti-human CXCR4 (CD184) or isotype control antibodies, and contour FACS plots analyzed cell surface expression of CXCR4. Harvested tumors generated from control and CXCR4 knockdown cells HT-29 (**G**) and DLD-1 (**H**) were subjected to Western blot analysis for CXCR4 and DR5. β-actin was used as protein loading control. Western Blot densitometric quantification numbers are shown above the loading control blot of all immunoblot studies.

A series of earlier in vitro experiments suggested that the CXCR4 protein but not its ligand-mediated signals is critical for CXCR4 mediated paclitaxel resistance. To validate our atypical in vitro observation into an in vivo system, we picked three different colon cancer cell lines having different CXCR4 expression patterns, such as HT-29, has robust CXCR4 surface expression and other two DLD-1 and HCT-116 having only intracellular CXCR4 expression. We inoculated 2 million control or CXCR4 knockdown cells of all three (HT-29, DLD-1 and HCT-116) into the flank of right or left hind leg of 4–6 weeks old nude Crl: CD1-Foxn1nu mice and assessed tumor progression. Remarkably, in all three xenograft animal models, irrespective of surface or intracellular expression of CXCR4, CXCR4 knockdown resulted in a significant reduction of tumor growth compared to their respective control (Fig. 5A–D and Supplementary Fig. S3, left panel). FACS analysis of single cells isolated from the respective tumors maintained their parental status of CXCR4 expression (Fig. 5E). CXCR4 surface expression was found to be almost nil in single cells isolated from both control and CXCR4 knockdown DLD-1 xenograft tumors (Fig. 5F). Western blot analysis of harvested tumors showed the marked upregulation of the expression of DR5 in the CXCR4 knockdown condition compared with their respective controls (Fig. 5G, H and Supplementary Fig. S3; right panel). Next, we used the HT-29 xenograft model and treated the animals with either vehicle control or paclitaxel for up to seven weeks. As shown in Supplementary Fig. S4, paclitaxel treatment significantly (*p* < 0.05) reduced the growth of tumors having CXCR4 knockdown cells as compared to control tumor-bearing animals treated with paclitaxel. Altogether, our in vivo studies in multiple xenograft settings suggest that CXCR4 total protein loss, regardless of its surface expression, result in compromised tumor growth, and chemotherapeutic drug paclitaxel can be even more effective to further sensitize these tumors under this condition.

### Increased intra-cellular CXCR4 expression enhances tumorigenesis and drug resistance in vitro and in vivo

**Fig. 6:**
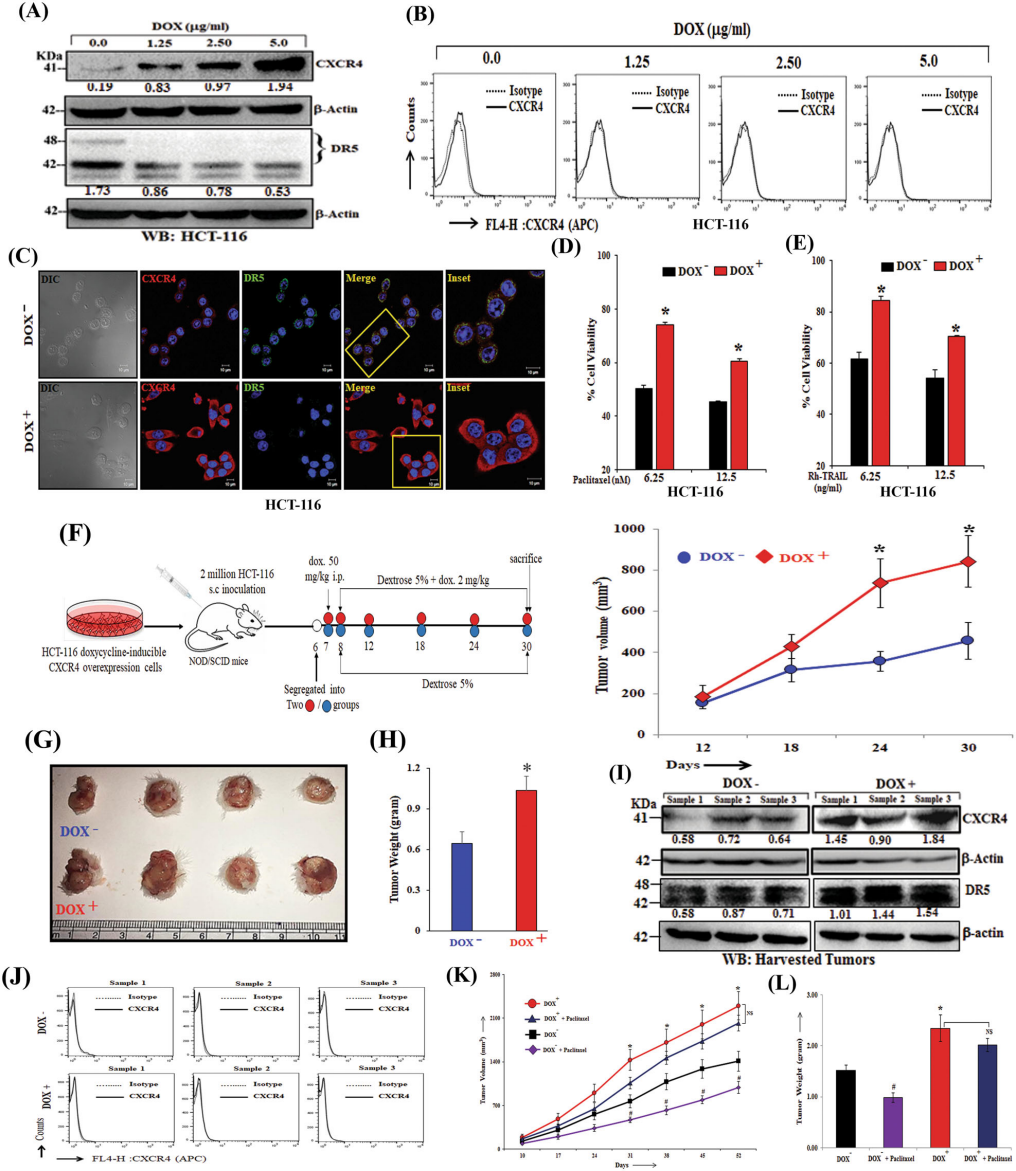
Intra-cellular CXCR4 overexpression promotes tumorigenesis and paclitaxel resistance in vitro and in vivo. **A** Western blot analysis of CXCR4 and DR5 in doxycycline-inducible CXCR4 overexpression HCT-116 cells after treatment with different concentrations of doxycycline (1.25 μg/ml, 2.50 μg/ml, 5 μg/ml) for 48 h. β-Actin was used as an internal protein loading control. **B** Doxycycline inducible CXCR4 overexpression HCT-116 cells were cultured under different concentrations of doxycycline (1.25 μg/ml, 2.5 μg/ml, 5 μg/ml) for 48 h. The cells were stained with APC-conjugated anti-human CXCR4 (CD184) antibody, and APC tagged IgG was used as isotype. The cells were analyzed by flow cytometry. Histogram overlays represent the surface expression level of CXCR4. **C** Doxycycline inducible CXCR4 overexpression HCT-116 cells were seeded on coverslips, treated with doxycycline (2μg/ml) for 48 h, subjected to immunofluorescence staining for CXCR4 as well as DR5 and analyzed by confocal microscopy. Inset photomicrographs represent the magnified area of the box. Scale bar, 10 µm. **D** Doxycycline inducible CXCR4 overexpression HCT-116 cells were treated with doxycycline (2 µg/ml) and Paclitaxel (6.25 nM, 12.5 nM) for 48 h and cytotoxicity was measured by SRB assay. Percent cell viability was tabulated. Columns, an average of triplicate readings of samples; error bars ± SEM. *p < 0.05 compared to uninduced cells. **E** Doxycycline inducible CXCR4 overexpression HCT-116 cells were treated with doxycycline (2 µg/ml) and TRAIL (6.25, 12.5 ng/ml) for 48 h and cytotoxicity was measured by SRB assay. Percent cell viability was tabulated. Columns, an average of triplicate readings of samples; error bars ± SEM. *p < 0.05 compared to un-induced cells. **F**–**G**, **H** In total, 2 × 10^6^ Doxycycline inducible CXCR4 overexpression HCT-116 cells in 100 μl PBS were injected subcutaneously in the right flank of 4–6 weeks old NOD/SCID mice respectively. Mice were fed with doxycycline (2 mg/ml, 5% dextrose in water). Tumor volumes were measured after regular intervals by using a digital caliper. Diagrammatic representation of experimental plan (**F**, left panel), tumor growth curve (**F**, right panel), harvested tumor pictures (**G**) and tumor weight bar graph (**H**) are shown. Results are reported as the mean ± SE. *p < 0.05 compared to vehicle-fed mice. **I** Western blot analysis of CXCR4 and DR5 in tumors harvested from the Dox^-^ and Dox^+^ mice. β-Actin was used as an internal protein loading control. Western Blot densitometric quantification numbers are shown above the loading control blot of all immunoblot studies. **J** Single cells were isolated from the Dox^+^ and Dox^−^ harvested tumors. The cells were either stained with APC-conjugated anti-human CXCR4 (CD184) antibody and PE-conjugated EpCAM (CD326) antibody or respective isotype control antibodies. The cells were analyzed by flow cytometry. Histogram overlays represent the surface expression level of CXCR4 in EpCAM positive HCT116 cell population. **K**, **L** In total, 5 × 10^6^ Doxycycline inducible CXCR4 overexpression HCT-116 cells in 100 μl PBS were injected subcutaneously in the flanks of the right or left hind leg of 4–6 weeks old NOD/SCID mice respectively. Mice were fed either with vehicle (5% dextrose in water) or doxycycline (2 mg/ml, 5% dextrose in water). Paclitaxel (5 mg/kg) was administered per week for 7 weeks. The tumor growth curve (**K**) and tumor weight (**L**) are shown, where points are indicative of the average value of tumor (*n* = 7) volume; mean ± SE. *p < 0.05 compared to vehicle-fed mice. ^#^*p* < 0.05 compared to vehicle-fed mice.

Next, we attempted to investigate the effect of CXCR4 overexpression on DR5 expression and drug resistance in vitro and in vivo. Using HCT-116 cells, we observed that these cells robustly express intracellular CXCR4 protein upon the induction of different doses of doxycycline treatment but failed to overexpress the same on the cell surface (Fig. 6A–C). This unusual property of HCT-116 cells allowed us to study the pro-tumorigenic functions of intracellular CXCR4 protein in more detail. Moreover, it was also observed that doxycycline induced CXCR4 overexpression at the intracellular level reduces the expression of DR5 (Fig. 6A) and exhibit significant (*p* < 0.01) resistance to paclitaxel (Fig. 6D) and TRAIL treatment (Fig. 6E). Next, 2 × 10^6^ HCT-116 doxycycline-inducible CXCR4 overexpression cells in PBS were injected subcutaneously in the right flank of the hind leg of 4–6 weeks old NOD/SCID mice. After the formation of palpable tumors, mice were randomized, grouped, and one of them fed with doxycycline in drinking water and observed for tumor progression for 24 days. As shown in Fig. 6F–H, doxycycline fed groups (CXCR4 overexpression) resulted in significant (*p* < 0.05) induction of tumor volume and weight as compared to the control group. We performed western blot analysis and observed marked induction of CXCR4 and less DR5 protein in the respective harvested tumors (Fig. 6I). Interestingly, FACS analysis of harvested tumor cells (CD326^+^ HCT116) did not display any induction of surface expression of CXCR4 in doxycycline group as compared to control (Fig. 6J) again suggesting that the promotion of tumor growth was due to the overexpression of intracellular CXCR4 protein. Further, we observe paclitaxel treatment significantly reduces tumor growth in control tumor bearing mice, whereas, failed to do so in CXCR4 overexpression condition in HCT-116 xenograft model (Figs. 6K, L). Altogether, these data indicate that upregulation of intracellular CXCR4 protein reduced the expression of DR5 and promoted tumorigenesis as well as resistance against chemotherapeutic drug paclitaxel in vitro and in vivo.

### Expression of CXCR4 and DR5 are inversely correlated in human breast cancer

**Fig. 7:**
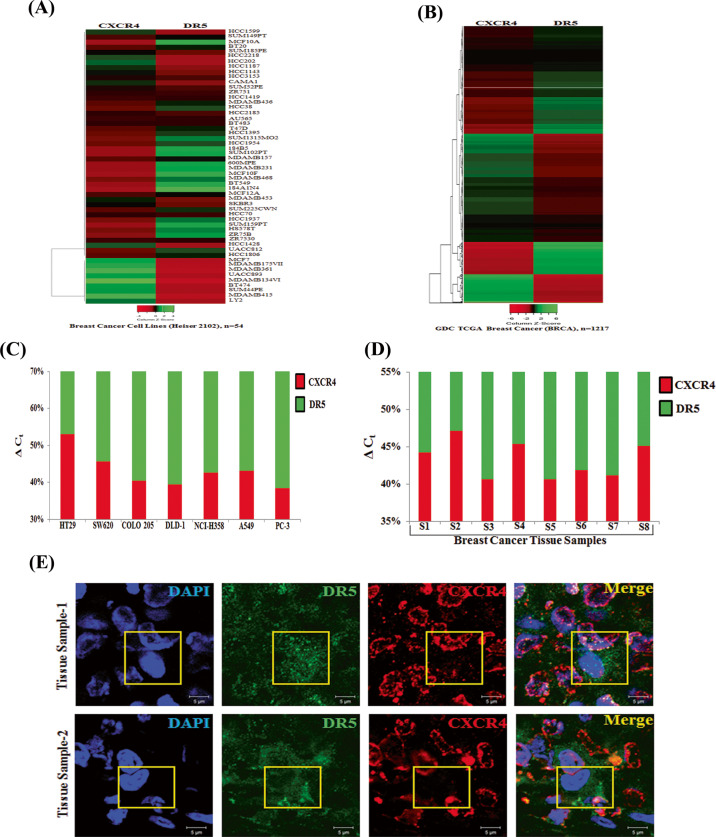
Expression of CXCR4 inversely correlates with DR5 expression in cancer cell lines, human breast cancer patient cohort and human breast cancer tissue samples. Heat maps are displaying CXCR4 and DR5 expression in (**A**) breast cancer cell lines (*n* = 54) and in (**B**) GDC TCGA Breast Cancer (BRCA) patient cohort (*n* = 1217). Shades of red and green represent expression values in fold change. Total RNA was isolated from various cancer cell lines (**C**) and breast cancer patient tumor tissue samples (**D**), reverse transcribed, and RT-qPCR was performed for CXCR4 and DR5 expression analysis. 18 s is used as an internal control. Percentage delta C_t_ was determined for each sample from quadruplicate C_t_ value and represented in bar graph having the differential contribution of CXCR4 (red) and DR5 (green) expression. **E** Human formalin-fixed paraffin-embedded mammary tumor tissues were subjected to immunofluorescence staining of CXCR4 (red) and DR5 (green) proteins and analyzed by confocal microscopy. Scale bar, 5 μm sections were viewed at 63X magnification. Yellow boxed merged confocal photomicrograph area represents cell positive for DR5 (green) staining having minimal CXCR4 (red) staining.

We exploited TCGA database to find out the clinical correlation between CXCR4 and DR5, especially focusing on breast cancer as it possesses large (*n* = 54) panel of breast cancer cell line data along with the significant amount of breast cancer patient (*n* = 1217) sample data^28^. First, we analyzed the expression profiles of CXCR4 and DR5 by using UCSC Xena (https://xena.ucsc.edu/) browser in a panel of breast cancer cell lines and GDC TCGA Breast Cancer (BRCA) patient cohort (Figs. 7A, B) and found inverse correlation between CXCR4 and DR5 expression in most of the breast cancer cell lines as well as in a large number of human breast cancer tissues. As panel cell line data is not available for human colon and other cancers, we further analyzed CXCR4 and DR5 expression by RT-qPCR in 7 different cancer cell lines, four of which belongs to colon cancer, and observed an inverse association between CXCR4 and DR5 expression in most of the solid tumor cell lines (Fig. 7C). Further, we evaluated the mRNA expression of CXCR4 and DR5 in human breast tissues by RT-qPCR, and found that in the majority of samples, CXCR4 expression is oppositely correlated with DR5 expression (Fig. 7D). Finally, by performing immunofluorescence microscopy, CXCR4 (red) protein expression was found to be predominant in human breast cancer tissues whereas, DR5 (green) expression was sparse (Fig. 7E). Altogether, these findings indicate that our in vitro observations are likely of pathophysiologic importance for the development of solid tumors, particularly in colon and breast cancer, where the inverse relationship between CXCR4 versus DR5 could be a critical indicator for predicting paclitaxel therapy response as well as the aggressiveness of the particular cancer.

## Discussion

The lineage of CXCR4, with its pro-tumorigenic functions in solid tumors, is unequivocal. Earlier publications suggest that the CXCR4-CXCL12 axis is not only pivotal for modulating cancer metastasis but also responsible for executing its other tumor-promoting functions^14,39,40^. Despite vast preclinical evidence, CXCR4 inhibitor ‘Plerixafor’ or AMD3100 got FDA approval as a stem cell mobilizer^41^, not as a cancer drug or even as a metastasis inhibitor. The discrepancy of preclinical observations and limited clinical success of CXCR4 antagonists as cancer therapy strongly advocates the involvement of some other aspects of CXCR4 biology beyond its classical CXCR4-CXCL12 signaling axis. Overexpression of multiple signaling impaired CXCR4 constructs suggests that intracellular presence of CXCR4 protein, but neither its surface presence nor CXCL12-CXCR4 mediated signals are essential in modulating DR5 expression and therapy (paclitaxel) resistance in cancer. Our results also justify to some extent why the CXCR4 inhibitor phenotype does not match with the CXCR4 null/knock-out phenotype under different preclinical settings.

Initially, we considered both CXCR4 and CXCR7 to study the impact of these two chemokine receptors to understand therapy resistance in cancer as they share CXCL12 as a common ligand, and are shown to be overexpressed in different solid tumors^14,17^. However, we observed selective involvement of CXCR4 in significantly modulating paclitaxel resistance in breast and colon cancers, which is actually in corollary with vast literature documenting the pro-tumorigenic role of CXCR4. Drug resistance is one of the identifying features of Cancer stem cells (CSCs)^25,42–44^ and CXCR4, being a bona fide CSC marker for prostate and pancreatic cancers^45,46^, evidently support its presence to promote therapy resistance. Also, studies in AML and NSCLC showed that the presence of CXCR4 has a positive correlation with therapy resistance though they consider ligand-mediated signals as a responsible reason for the same^47,48^. Our unbiased mechanistic hunt discovered that CXCR4 mediates therapy resistance via selectively regulating the pro-apoptotic candidate protein DR5 by differentially modulating YY1 and p53 recruitment at the promoter site of the DR5 gene. YY1 recruitment in the promoter region for repression of target genes is documented by earlier elegant studies^49,50^. Further, CXCR4-YY1 reciprocal regulation has been well documented as a pro-tumorigenic function of CXCR4 in AML therapy resistance and osteosarcoma angiogenesis^47,51^. CXCR4 is rarely found to be localized in the nucleus, which is linked with poor prognosis and enhanced metastasis^52–54^. Nuclear localization of CXCR4 may be associated with transcriptional upregulation of YY1 and its differential recruitment to the promoter region of the *DR5* gene. Though CXCR4 mediated DR5 transcriptional regulation is our novel finding, it has been reported in the studies that high CXCR4 expression in the cancer cells is correlated with poor prognosis and resistance against the various DNA damaging chemotherapeutic agents whose mechanism of action involve the regulation of Death receptors^55,56^.

Utilizing three different xenograft models of colon cancer cells that are either expressing surface CXCR4 (HT-29) or are null for the CXCR4 surface expression (DLD-1, HCT-116), we provided strong evidence that knockdown of CXCR4 results in reduced tumor growth and paclitaxel sensitization irrespective of their surface expression status (Fig. 5 and Fig. S1). In support of our in vivo observations, several previous studies have demonstrated that CXCR4 knockdown cells produce smaller tumors as compared to their control counterparts^40,57,58^. Interestingly, at least one study indicated that the cytoplasmic expression of CXCR4 is correlated with tumor burden and the metastatic load of certain cancers^59^. Further, some reports have suggested that the in vivo environment gives cues to the cancer cells to transport their intracellular CXCR4 on the surface^39,60^, so to test the same, we isolated single cells from harvested in vivo xenograft tumors and examined the CXCR4 surface expression. However, no change was found in the surface expression of CXCR4 either in control or the knockdown cells suggesting the fact that though they gave rise to smaller tumors compared to control, there is no contribution of CXCR4-CXCL12 signaling axis in delivering this phenotype. Inverse correlation of CXCR4 and DR5 expression was observed in human breast cancer samples, which was primarily allied with TCGA data obtained from a broad panel of breast cancer cell lines as well as data from human TCGA Breast Cancer (BRCA) cohort suggesting the clinical significance of our finding.

Overall, the study indicates that high levels of CXCR4 intracellular protein but not CXCR4 signaling restricts cellular apoptosis and promotes cell survival via downregulating the DR5 expression and thus renders cancer cells resistant to chemotherapeutic drugs like paclitaxel. Further, intracellular CXCR4 protein contributes significantly to the tumorigenic potential of cancer cells, and future therapies should majorly focus on targeting CXCR4 protein and not only CXCR4 mediated signals. Targeting selectively CXCR4 protein in cancer cells would not be easy. However, our findings open up a huge possibility for the further discovery of CXCR4 interacting protein partners, which might have an immense role in posing its pro-tumorigenic effect and can be effectively targeted by small molecule inhibitors. Moreover, our original finding may trigger the discovery of the novel intracellular role of other chemokine receptors in the context of particular disease pathophysiology, which has been overlooked so far.

## Supplementary information

Supplementary Data

## Data Availability

All the data needed to evaluate the conclusions in the paper are present in the paper and/or as Supplementary data. Additional data related to this paper will be made available upon reasonable request.
