# Supplementary material for: CXCR4 intracellular protein promotes drug resistance and tumorigenic potential by inversely regulating the expression of Death Receptor 5
Source: Cell Death Dis. 2021 May 8;12(5):464. doi: 10.1038/s41419-021-03730-8 (PMC8106681; doi:10.1038/s41419-021-03730-8)
Supplement: Supplementary file 1 — Supplementary Data [file 41419_2021_3730_MOESM1_ESM.pdf]

Supplementary Data

CXCR4 intracellular protein promotes drug resistance and tumorigenic potential by inversely regulating the expression of Death Receptor 5 by *Nengroo et.al.,*

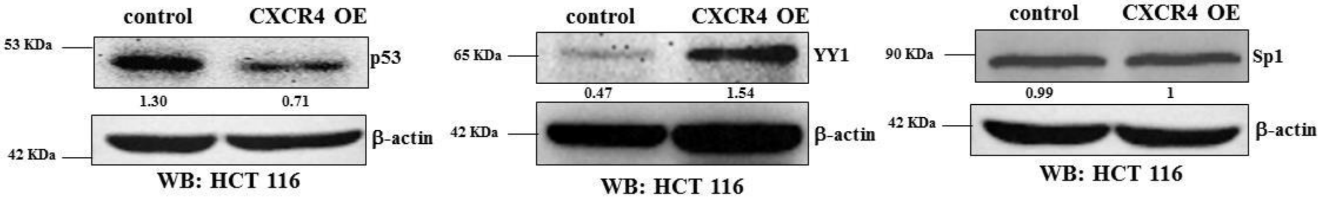

Supplementary Figure S1: CXCR4 overexpression results in down and upregulation of p53 and YY1 respectively in HCT-116 cells.
Western blot analysis of p53, YY1, and Sp1 in control and CXCR4 overexpressing HCT-116 cells; β-actin was used as the protein loading control. Western Blot densitometric quantification numbers are shown above the loading control blot of all immunoblot studies.

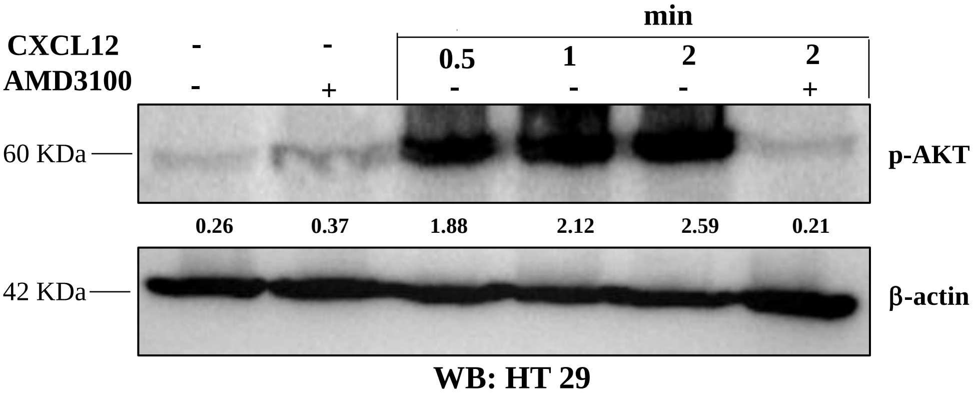

Supplementary Figure S2: Effect of AMD3100 and CXCL12 on the phosphorylation of AKT in HT-29 cells
HT-29 cells were either pre-treated with vehicle or AMD3100 (5 μM) for 12 hours, followed by treatment with CXCR4 ligand CXCL12 (100ng/ml) for different time points (0.5, 1 and 2 mins) and subjected to Western blot analysis for p-AKT and β-actin. Western Blot densitometric quantification numbers are shown above the loading control blot of all immunoblot studies.

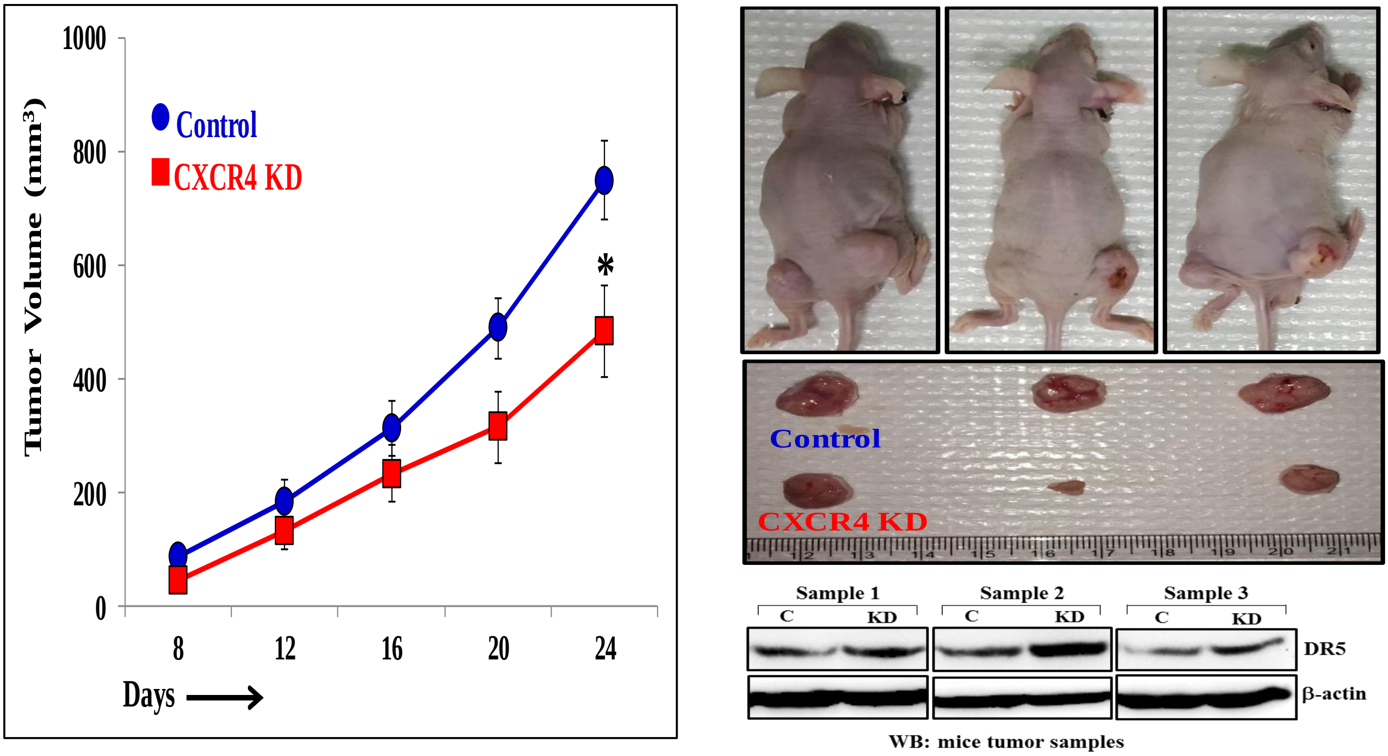

Supplementary Figure S3: CXCR4 knockdown results in compromised tumor growth and DR5 overexpression *in vivo* in HCT-116 xenograft model.

2 million stable control or CXCR4 knockdown HCT-116 cells in 100 ml PBS were injected subcutaneously in the flanks of the right or left hind leg of 4-6 weeks old Crl:CD1-Foxn1nu mice. Tumor volumes were measured after regular intervals by using a caliper. Tumor growth curves are shown; points are indicative of average value of tumor volume (n=10); bars, ± SE. *$p<0.05$ compared to control tumors. Middle panel shows representative images of tumor bearing mice, control (right flank) and CXCR4 knock down (left flank). Mice were sacrificed and respective tumors were harvested and shown in photographs. Harvested tumors generated from control and CXCR4 knockdown cells were subjected to Western blot analysis for DR5 and β-actin (lower panel)

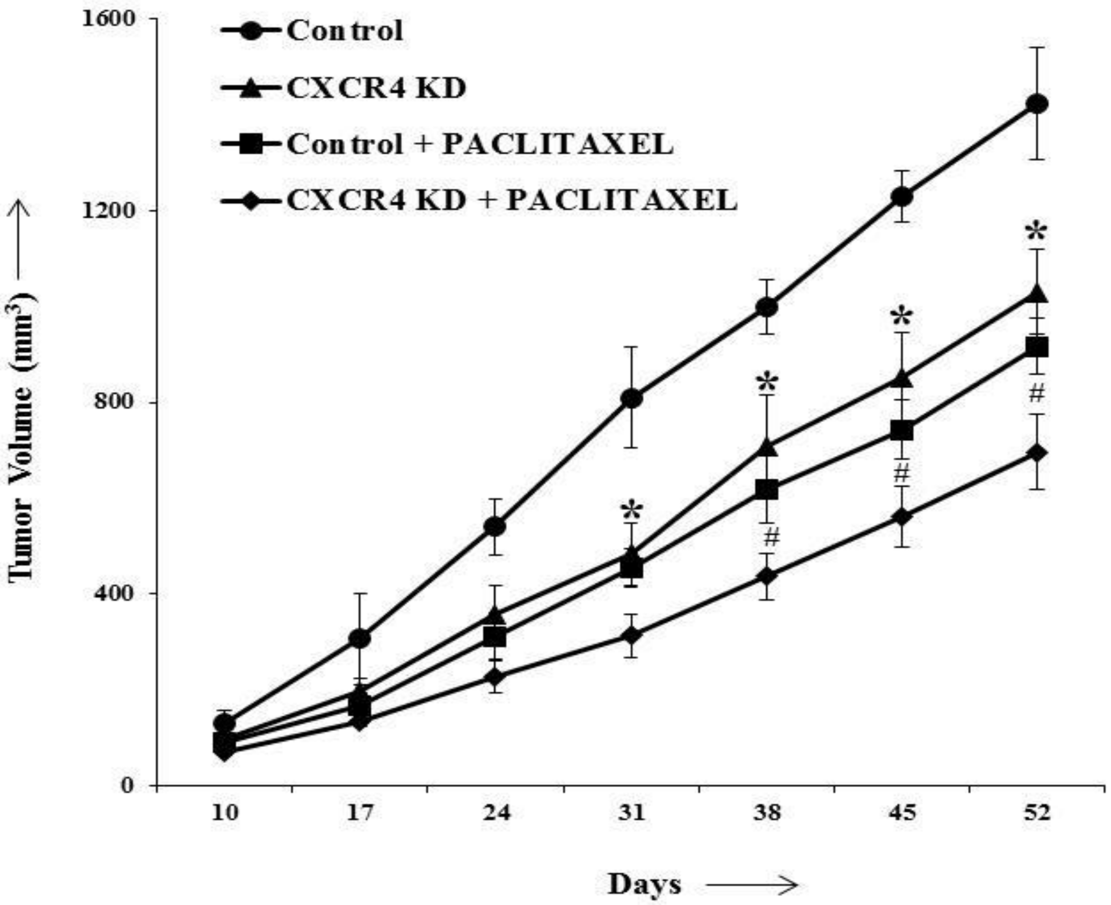

Supplementary Figure S4: CXCR4 knockdown results in paclitaxel sensitization *in vivo*

5 million stable control or CXCR4 knockdown HT-29 cells in 100 μl PBS were injected subcutaneously in the flanks of the right or left hind leg of 4-6 weeks old Crl: CD1-Foxn1nu mice respectively. Paclitaxel (5mg/kg) was administered to both groups intra-peritoneally per week for seven weeks. Tumor volumes were measured after regular intervals by using a digital caliper. Tumor growth curves for control (filled circle), control + Paclitaxel (filled square), CXCR4 KD (filled triangle), and CXCR4 KD + Paclitaxel (filled rhombus) are shown; points are indicative of the average value of tumor volume (n=7); bars, ± SE. *$p<0.05$ compared to control groups, $^{\#}p<0.05$ compared to control + paclitaxel group.

CXCR4 mutation confirmation by Sanger sequencing

The confirmed sequences after the respective mutation are as:

L86P substitution mutation:

ATGGAGGGGATCAGTATATACACTTCAGATAACTACACCGAGGAAATGGGCTCAGGGGACTATGACTCCATGA
AGGAACCCTGTTTCCGTGAAGAAAATGCTAATTTCAATAAAATCTTCCTGCCCACCATCTACTCCATCATCTTCTT
AACTGGCATTGTGGGCAATGGATTGGTCATCCTGGTCATGGGTTACCAGAAGAAACTGAGAAGCATGACGGA
CAAGTACAGGCTGCACCTGTCAGTGGCCGACCTC**CCT**TTTGTCATCACGCTTCCCTTCTGGGCAGTTGATGCCGT
GGCAAACTGGTACTTTGGGAACTTCCTATGCAAGGCAGTCCATGTCATCTACACAGTCAACCTCTACAGCAGTG
TCCTCATCCTGGCCTTCATCAGTCTGGACCGCTACCTGGCCATCGTCCACGCCACCAACAGTCAGAGGCCAAGG
AAGCTGTTGGCTGAAAAGGTGGTCTATGTTGGCGTCTGGATCCCTGCCCTCCTGCTGACTATTCCCGACTTCATC
TTTGCCAACGTCAGTGAGGCAGATGACAGATATATCTGTGACCGCTTCTACCCCAATGACTTGTGGGTGGTTGT
GTTCCAGTTTCAGCACATCATGGTTGGCCTTATCCTGCCTGGTATTGTCATCCTGTCCTGCTATTGCATTATCATC
TCCAAGCTGTCACACTCCAAGGGCCACCAGAAGCGCAAGGCCCTCAAGACCACAGTCATCCTCATCCTGGCTTT
CTTCGCCTGTTGGCTGCCTTACTACATTGGGATCAGCATCGACTCCTTCATCCTCCTGGAAATCATCAAGCAAGG
GTGTGAGTTTGAGAACACTGTGCACAAGTGGATTTCCATCACCGAGGCCCTAGCTTTCTTCCACTGTTGTCTGA
ACCCCATCCTCTATGCTTTCCTTGGAGCCAAATTTAAAACCTCTGCCCAGCACGCACTCACCTCTGTGAGCAGAG
GGTCCAGCCTCAAGATCCTCTCCAAAGGAAAGCGAGGTGGACATTCATCTGTTTCCACTGAGTCTGAGTCTTCA
AGTTTTCACTCCAGCTAA

Deletion mutation (amino acid residues 242-248):

ATGGAGGGGATCAGTATATACACTTCAGATAACTACACCGAGGAAATGGGCTCAGGGGACTATGACTCCA
TGAAGGAACCCTGTTTCCGTGAAGAAAATGCTAATTTCAATAAAATCTTCCTGCCCACCATCTACTCCATCA
TCTTCTTAACTGGCATTGTGGGCAATGGATTGGTCATCCTGGTCATGGGTTACCAGAAGAAACTGAGAAG
CATGACGGACAAGTACAGGCTGCACCTGTCAGTGGCCGACCTCCTCTTTGTCATCACGCTTCCCTTCTGG
GCAGTTGATGCCGTGGCAAACTGGTACTTTGGGAACTTCCTATGCAAGGCAGTCCATGTCATCTACACAG
TCAACCTCTACAGCAGTGTCCTCATCCTGGCCTTCATCAGTCTGGACCGCTACCTGGCCATCGTCCACGCC
ACCAACAGTCAGAGGCCAAGGAAGCTGTTGGCTGAAAAGGTGGTCTATGTTGGCGTCTGGATCCCTGCC
CTCCTGCTGACTATTCCCGACTTCATCTTTGCCAACGTCAGTGAGGCAGATGACAGATATATCTGTGACCG
CTTCTACCCCAATGACTTGTGGGTGGTTGTGTTCCAGTTTCAGCACATCATGGTTGGCCTTATCCTGCCTG
GTATTGTCATCCTGTCCTGCTATTGCATTATCATCTCCAAGCTGTCACACTCCAAGGGCCACCAGAAGCGCA
AGGCCCTCAAGACCAC**AT**TCGCCTGTTGGCTGCCTTACTACATTGGGATCAGCATCGACTCCTTCATCCTC
CTGGAAATCATCAAGCAAGGGTGTGAGTTTGAGAACACTGTGCACAAGTGGATTTCCATCACCGAGGCC
CTAGCTTTCTTCCACTGTTGTCTGAACCCCATCCTCTATGCTTTCCTTGGAGCCAAATTTAAAACCTCTGCCC
AGCACGCACTCACCTCTGTGAGCAGAGGGTCCAGCCTCAAGATCCTCTCCAAAGGAAAGCGAGGTGGA
CATTCATCTGTTTCCACTGAGTCTGAGTCTTCAAGTTTTCACTCCAGCTAA
